# Self‐Assembled Moiré Superlattices of Ti_3_C_2_T*_x_* MXene for Future Twistronic Applications

**DOI:** 10.1002/advs.202504394

**Published:** 2025-07-30

**Authors:** Kuanysh Zhussupbekov, Andrea Cabero del Hierro, Samuel Berman, Dahnan Spurling, Ainur Zhussupbekova, Stefano Ippolito, David D. O'Regan, Igor Shvets, Yury Gogotsi, Valeria Nicolosi

**Affiliations:** ^1^ School of Physics Trinity College Dublin The University of Dublin Dublin D02 PN40 Ireland; ^2^ School of Chemistry Trinity College Dublin The University of Dublin Dublin D02 PN40 Ireland; ^3^ A.J. Drexel Nanomaterials Institute and Department of Materials Science and Engineering Drexel University Philadelphia PA 19104 USA; ^4^ Centre for Research on Adaptive Nanostructures and Nanodevices (CRANN) and Advanced Materials and Bioengineering Research (AMBER) Trinity College Dublin The University of Dublin Dublin D02 PN40 Ireland

**Keywords:** DFT, electronic structure, Moiré superlattice, MXenes, STM, STS

## Abstract

Nanoscale periodic Moiré superlattices based on 2D heterostructures offer an opportunity to unveil and exploit electronic and quantum properties that are not present in single‐layer 2D and/or 3D bulk counterparts. However, a detailed understanding of the Moiré superlattices and their resulting electronic structure at the atomic scale is currently lacking in many systems, such as the fastest‐growing family of 2D materials, MXenes. This is crucial for gaining fundamental knowledge and mastery over quantum phenomena in these materials. This study thoroughly examines and compares the self‐assembled Moiré superlattices of the most prominent MXene, Ti_3_C_2_T*_x_*, by combining experimental scanning tunneling microscopy and spectroscopy with density functional theory calculations. Three distinct self‐assembled Moiré patterns with a periodicity of 2.52, 2.39, and 1.25 nm are investigated. Experimental and theoretical data reveal that the Moiré superlattice with a periodicity of 1.25 nm exhibits a spatial modulation of the density of states in the conduction band due to electronic interlayer coupling effects. The findings unveil MXene Moiré superlattices at the atomic level and pave the way to a new research field in MXetronics and twistronics with great potential for quantum devices and related applications.

## Graphical abstract

Scanning tunneling microscopy imaging reveals self‐assembled Moiré superlattices in MXene with distinct periodicities. The observed Moiré modulates the local density of states due to interlayer electronic coupling. This work provides an atomic‐scale understanding of MXene Moiré physics and establishes a foundation for twistronics in future quantum devices.

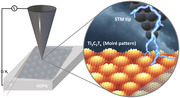

## 1 Introduction

2D materials exhibit different chemical and physical properties compared to their bulk counterparts.^[^ ^1^, ^2^ ^]^ When two monolayers of van der Waals materials, such as graphene or MoS_2_, are stacked vertically with a twist angle or lattice mismatch, they may create a Moiré superlattice due to the long‐wavelength periodic potential arising from the interlayer atomic interactions.^[^ ^3^, ^4^, ^5^, ^6^, ^7^, ^8^ ^]^ The change of the twist angle can generate Moiré superlattices with different periodicity, which allows for the tuning of the electronic properties due to the changes in the band structure of the material.^[^ ^9^, ^10^, ^11^, ^12^, ^13^ ^]^ Recently, superconducting and insulating properties were discovered in twisted bilayer graphene at the so‐called “magic” angle.^[^ ^6^, ^14^ ^]^ Therefore, gaining insight into the electron‐electron interactions in a Moiré superlattice is paramount for understanding their unique quantum properties and developing innovative applications in 2D materials science.^[^ ^6^, ^14^, ^15^, ^16^ ^]^ Although 2D transition metal carbides, nitrides, and carbonitrides (also known as MXenes) are the latest, largest, and fastest‐growing family of 2D materials, no investigations on their Moiré superlattices have been reported to date, leaving a major gap in the understanding of their electronic properties and quantum phenomena.

Our work focuses on the investigation of self‐assembled Ti_3_C_2_T*_x_* MXene Moiré superlattices. Ti_3_C_2_T*_x_* is the first‐discovered and most studied member of the MXene family.^[^ ^17^ ^]^ MXenes have the chemical formula M*_n_*_+1_X*_n_* T*_x_*, where *n* ranges from 1 to 4, M stands for an early transition metal, X is carbon and/or nitrogen, and T*_x_* describes their surface terminations (typically oxygen, hydroxyl groups, halogens, and/or chalcogens). The surface chemistry strongly affects their mechanical, electronic, and optical properties.^[^ ^18^, ^19^, ^20^, ^21^, ^22^, ^23^, ^24^ ^]^ Scanning tunneling microscopy (STM) and scanning tunneling spectroscopy (STS) were only recently used to characterize the surface of MXene samples.^[^ ^25^ ^]^ STM is widely employed in surface science, and it exploits the quantum tunneling principle to obtain images with atomic resolution.^[^ ^26^ ^]^ STS can also be performed using STM equipment, with the main objective being to examine the local density of states (LDOS). As the differential conductance *dI/dV* (where *I* refers to the tunneling current flowing between the STM tip and the sample surface and *V* refers to the applied bias voltage) is generally proportional to the LDOS, STS measurements are able to assess whether the material is metallic, semi‐metallic, or semiconducting.^[^ ^26^ ^]^ Both techniques are extensively used as they provide high‐quality atomic resolution and electronic information, while being non‐destructive during scanning.^[^ ^27^ ^]^ For such reasons, STM and STS are employed in the investigation of Moiré superlattices.^[^ ^28^ ^]^ In fact, several studies have determined structural information from Moiré superlattices on various materials using STM and used STS to explore their singular electronic properties.^[^ ^29^, ^30^, ^31^, ^32^ ^]^ To the best of our knowledge, the present study is the first to report on three distinct self‐assembled Moiré superlattices of MXenes, also using density functional theory (DFT) to calculate their electronic properties as well as provide support to the interpretation and explanation of experimental results.

## 2 Results

**Figure 1:**
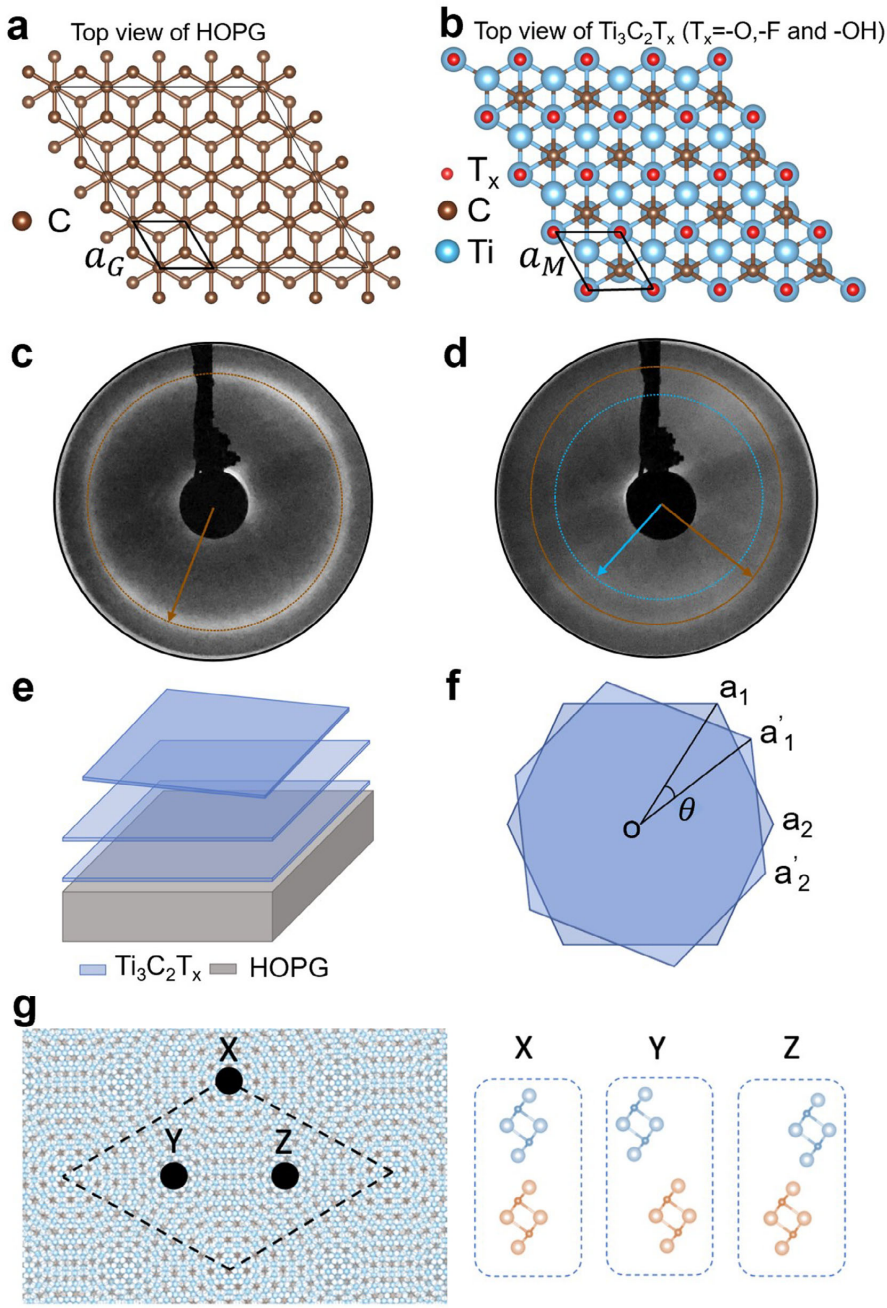
Structural models and LEED patterns. a) Structural model of HOPG in top view. b) Structural model of Ti_3_C_2_T*_x_* in top view. c,d) LEED pattern of HOPG surface cleaved in UHV and after drop‐casting of MXene at a primary beam energy *E*_p_ = 118 eV. e) Schematic representation of MXene layers on top of HOPG substrate. f) Layer rotation of MXene flakes. g) Model of the simulated Moiré pattern, showing *AA* stacking with the corresponding high symmetry points (left side). Alignment of MXene layers at the high symmetry points X, Y, and Z, where large circles are titanium atoms and small circles are carbon atoms (the right side shows the cross‐section perpendicular to the surface of the left side image).

The biggest challenge of STM/S studies lies in the difficulty in obtaining large and high‐quality MXene single crystals, limiting the examination of their electronic properties. In this investigation, we addressed this problem by drop‐casting a Ti_3_C_2_T*_x_* aqueous dispersion in an inert atmosphere onto a freshly cleaved surface (under ultra‐high vacuum (UHV) conditions) of highly ordered pyrolytic graphite (HOPG). Upon deposition, vacuum drying was performed, producing single‐layer MXene flakes on HOPG, with many flakes overlapping randomly under different rotation angles. More details on the surface preparation procedure are provided in the Methods section. The structural models of HOPG and Ti_3_C_2_T*_x_* are illustrated in **Figure** **1**a,b. The successful surface preparation was confirmed by low‐energy electron diffraction (LEED). Figure 1c shows the LEED pattern of the cleaved HOPG in UHV, with the strong circular reflex (brown dotted circle) indicating the polycrystalline nature of HOPG with a single lattice constant. In contrast, Figure 1d presents the LEED pattern after drop‐casting MXene from a solution with a concentration of 0.01 mg mL^−1^ under an N_2_ atmosphere. A second circular reflex (blue dotted circle) appears in the latter LEED pattern, corresponding to the larger lattice constant of the MXene in real space. The two circular reflexes in Figure 1d indicate the presence of two materials at the surface. The results were verified by a lattice constant ratio calculation, based on the experimental LEED images as well as considering literature values.^[^ ^33^, ^34^ ^]^ The lattice constant ratio calculated from LEED was 1.264, while the ratio calculated using literature values was 1.244 (Figure SM2, Supporting Information), indicating a good agreement. Additionally, X‐ray photoelectron spectroscopy (XPS) was used to study the composition and surface purity of the vacuum‐dried MXene (Figure SM3, Supporting Information).

Figure 1e shows a simplified representation of MXene flakes (blue sheets) on top of the HOPG substrate (grey parallelepiped), while a schematic illustration of the Moiré pattern is displayed in Figure 1f, where the two topmost layers are rotated at an angle *θ*. The Moiré corrugation observed in the STM images results from different *AA* high‐symmetry stackings depicted in Figure 1g. The black dashed line (left side) shows the supercell with three specific locations (viz., high symmetry points) designated as *X*, *Y*, and *Z*, whose side‐view alignments (cross‐section perpendicular to the surface) are shown on the right side. In such alignments, the large empty circles represent Ti atoms, while the small circles represent C atoms.

**Figure 2:**
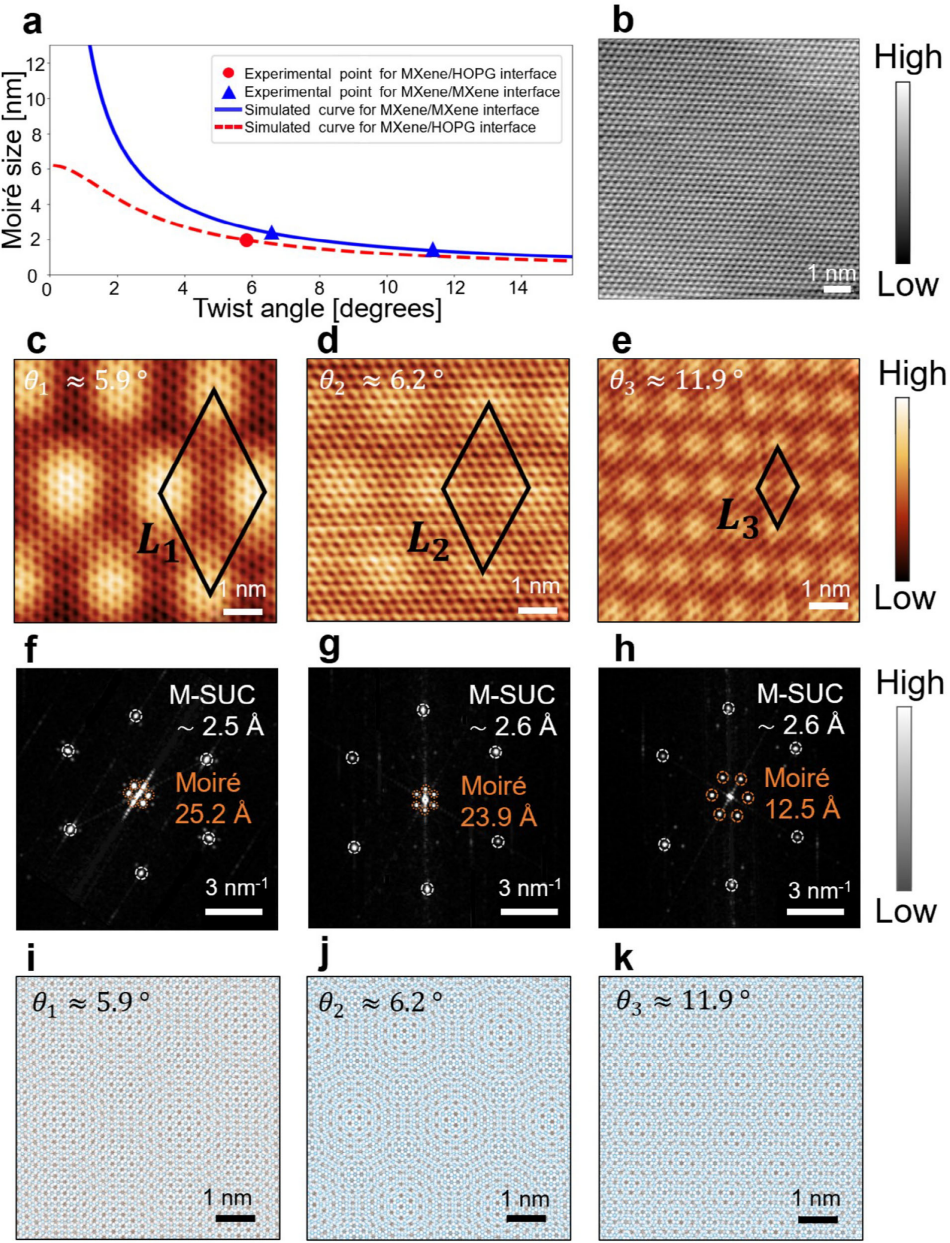
STM and FFT analysis. a) Dependence of Moiré size on the twist angles between the interfaces. The red dot corresponds to the experimental point for the MXene/HOPG interface. The red dashed line is the simulated curve for the MXene/HOPG interface. The blue triangles correspond to the experimental points for the MXene/MXene interface. The blue curve is the simulated curve for the MXene/MXene interface. b) STM topographic image of HOPG with atomic resolution (10 × 10 nm, V = 500 mV and I = 60 pA). c–e) STM topographic images showing Moiré patterns at different angles (*θ*_1_ = 5.9°, *θ*_2_ = 6.2°, and *θ*_3_ = 11.9°, from left to right) with the black rhombuses showing the supercell. c) STM topographic image of MXene/HOPG interface (7 × 7 nm, V = −1.2 V and I = 291 pA), d) STM topographic image of MXene/MXene interface (7 × 7 nm, V = −1.2 V and I = 291 pA), and e STM topographic image of MXene/MXene interface (7 × 7 nm, V = 1.5 V and I = 555 pA). f–h) Fast Fourier transform of the STM topographic images in c, d, and e, where M‐SUC corresponds to the MXene single unit cell. i–k) Model of simulated Moiré patterns (7 × 7 nm) of the STM topographic images in c, d, and e.

STM was performed on the sample to reveal the atomic structure of MXene and identify Moiré patterns. This resulted in the observation of three different self‐assembled Moiré superlattices, as displayed in **Figure** **2**c–e. Figure 2b shows an experimental STM topographic image revealing the atomic structure of HOPG, while Figures 2c–e display the Moiré patterns at different angles, namely *θ*_1_ = 5.9°, *θ*_2_ = 6.2°, and *θ*_3_ = 11.9°. The black rhombuses in the STM images (Figure 2c–e) are labelled *L*_1_, *L*_2_, and *L*_3_ and display the supercell of the Moiré patterns in real space. It should be noted that the Moiré structure with *L*_1_ supercell represents the interface between HOPG and MXene, while Moiré structures with *L*_2_ and *L*_3_ represent MXene/MXene interfaces. The effect of HOPG or the underlying Moiré might impact the top Moiré to some extent. However, STM is a surface‐sensitive technique that provides insights into the electronic properties of the top‐most Moiré pattern. Thus, the effect of the HOPG or underlying Moiré patterns is likely minimal. Details of these interfaces (individual STM images with line profiles) are discussed in the Supporting Information (Figure SM4, Supporting Information). To calculate the length of the Moiré supercell, the fast Fourier transform (FFT) of the STM topographic image was computed. Therefore, three different self‐assembled Moiré patterns with periods of approximately *L_1_* = 2.52, *L_2_* = 2.39, and *L_3_* = 1.25 nm were recorded. The Moiré corrugation observed in the STM images results from different stackings of two layers with hexagonal symmetry (Figure 2c–e). We designated specific high symmetry locations as *X*, *Y*, and *Z*, and these stackings are depicted in Figure 1g based on structural modelling. Figure 2f–h shows the FFTs of the STM topographic images in Figure 2c–e, respectively. These FFTs demonstrate a clear Moiré representation in reciprocal space, where M‐SUC represents the single unit cell of MXene, whose values are in good agreement with previous high‐resolution transmission electron microscopy reports.^[^ ^35^, ^36^ ^]^ The STM topographic images in Figure 2c–e were compared to the simulated model of Moiré patterns in real space shown in Figure 2i–k. The calculations of the angles for the Moiré areas are discussed in the Supporting Information. Two methods were used to calculate the angles: the first method utilises an equation to obtain the angle as illustrated by Wang J. et al,^[^ ^37^ ^]^ while the second method employs FFT (see Figure SM1, Supporting Information) to extract the twist angles as demonstrated by Zhao, W.M. et al.^[^ ^4^ ^]^ We show the atomic structure of the Moiré patterns at different angles separately in real and reciprocal space, providing an excellent agreement between simulated and experimental results for the superlattices. Moreover, the Moiré angles calculated using the STM topographic images were compared to the simulated curves predicted by the equations used in the method described in the Supporting Information. Figure 2a shows the dependence of the Moiré structure size on the twist angles between the MXene/MXene and MXene/HOPG interfaces. The red dot in the graph corresponds to the experimental point for the MXene/HOPG interface at a *θ*_1_ = 5.9° angle, while the red dashed line is a simulated curve for the MXene/HOPG interface obtained from Equation (1) in the Supporting Information. The blue triangles correspond to the experimental points observed by STM for the MXene/MXene interface at a *θ*_2_ = 6.2° and *θ*_3_ = 11.9° angle, respectively, while the blue curve is the simulated curve for the MXene/MXene interface obtained by Equation (4) in the Supporting Information. The experimental points are close to their corresponding simulated curves, corroborating that the method to predict the angles from the STM topographic images is accurate.

**Figure 3:**
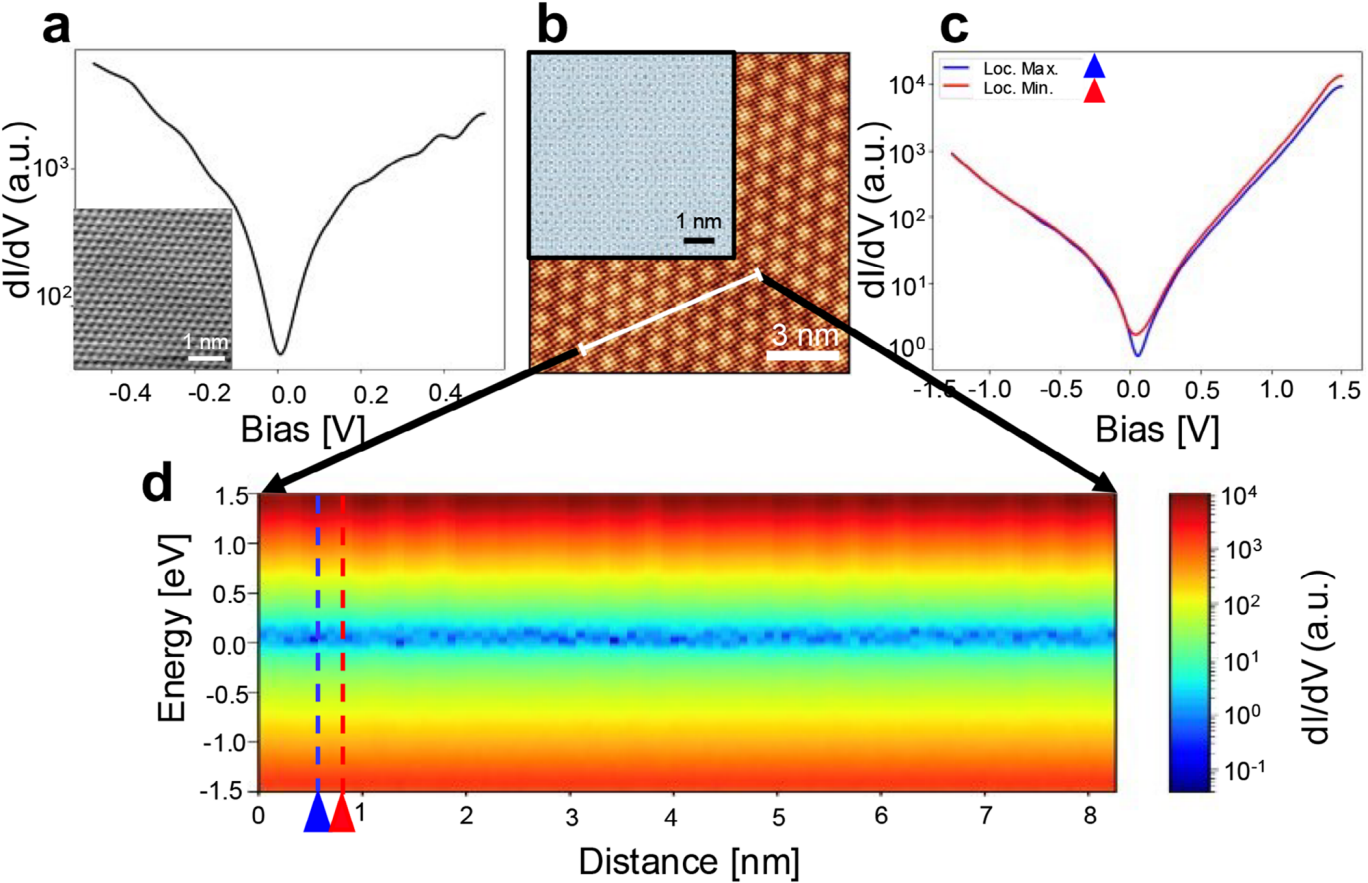
STS investigation of the Moiré pattern. a) Representative *dI/dV* spectra on a log scale, with STM image of HOPG surface as inset. b) STM topographic image, with the simulated Moiré pattern as inset, of the MXene/MXene interface as Figure 2e. c) Representative *dI/dV* spectra on dark and light oscillations in the log scale. d) 2D plot of conductance spectra on a log scale across the white line (*dI/dV* in the bias range of ±1.5 V), which demonstrates the oscillations along the white line in b.

To shed light on the electronic structure and properties of the MXene Moiré superlattice, STS measurements were performed. **Figure** **3**a shows representative *dI/dV* spectra of the clean HOPG surface, where the inset is the atomically resolved STM image of the HOPG. Figure 3b is the topographic STM image of the MXene/MXene interface at the *θ*_3_ angle, the same area as Figure 2e, and the inset displays the simulated Moiré pattern at the same angle.

STS measurements were conducted at each of the 100 points along the 8.25 nm length of the white line shown in Figure 3b.^[^ ^38^ ^]^ *I*–*V* curves were measured for each individual point and subsequently differentiated with respect to voltage.^[^ ^39^ ^]^ Figure 3c displays two representative *dI/dV* spectra taken at different points. Overall, LDOS looks homogenous in space over the entire energy range, except from 0.4 to 1.5 eV (empty states), where we observed a noticeable modulation of the LDOS (Figure 3c). The blue curve, marked by a blue triangle in Figure 3d, was acquired at point 8 and represents a local minimum in the LDOS. In contrast, the red curve, marked by a red triangle at point 12, corresponds to a local maximum in the LDOS. The resultant *dI/dV* profiles are depicted in Figure 3d using a 2D representation: each vertical line stands for a *dI/dV* profile, spanning from −1.5 to +1.5 V, with the intensity of *dI/dV* indicated using a spectrum of colours where warmer shades denote greater values. Measurements were performed every ≈0.8 Å along the white line in Figure 3b. The spatial transformation of the Moiré superlattice of the MXene is visualized through the 2D map, whose influence on the electronic structure is noteworthy. The spatial modulation of the local density of states observed in Moiré superlattice of the MXene within the 0.4–1.5 eV energy range arises primarily from the aperiodic variation in local atomic registry (i.e., the relative alignment between atoms in different layers), the resulting long‐range variation in the external potential (and thereby also the internal potential of the DFT Kohn‐Sham system). In other words, interlayer coupling (ordinarily weak but adding up over the complete Moiré superlattice) is understood to be responsible as the primary physical mechanism. It is known from previous studies that in such Moiré structures, the local stacking configuration changes across the unit cell, leading to electronic effects including site‐dependent modifications in potential energy, orbital overlap, and charge redistribution, like in TMDs.^[^ ^40^, ^41^, ^42^ ^]^ These variations modulate the local band structure and electronic coupling, giving rise to a periodic LDOS contrast observable via STS.

DFT calculations were performed with the aim of obtaining a better understanding of the experimental results and were conducted by constructing a supercell that closely approximates the periodicity of the experimental STM images, as illustrated in Figure 2c–e, and by assuming only the two topmost layers were responsible for the formation of the Moiré pattern. The calculations were only carried out for the structure with the angle of *θ*_3_ as shown in Figure 2e, which has the smallest supercell. The calculations for Figure 2c,d were not computed due to the extremely large dimensions of the supercell.

**Figure 4:**
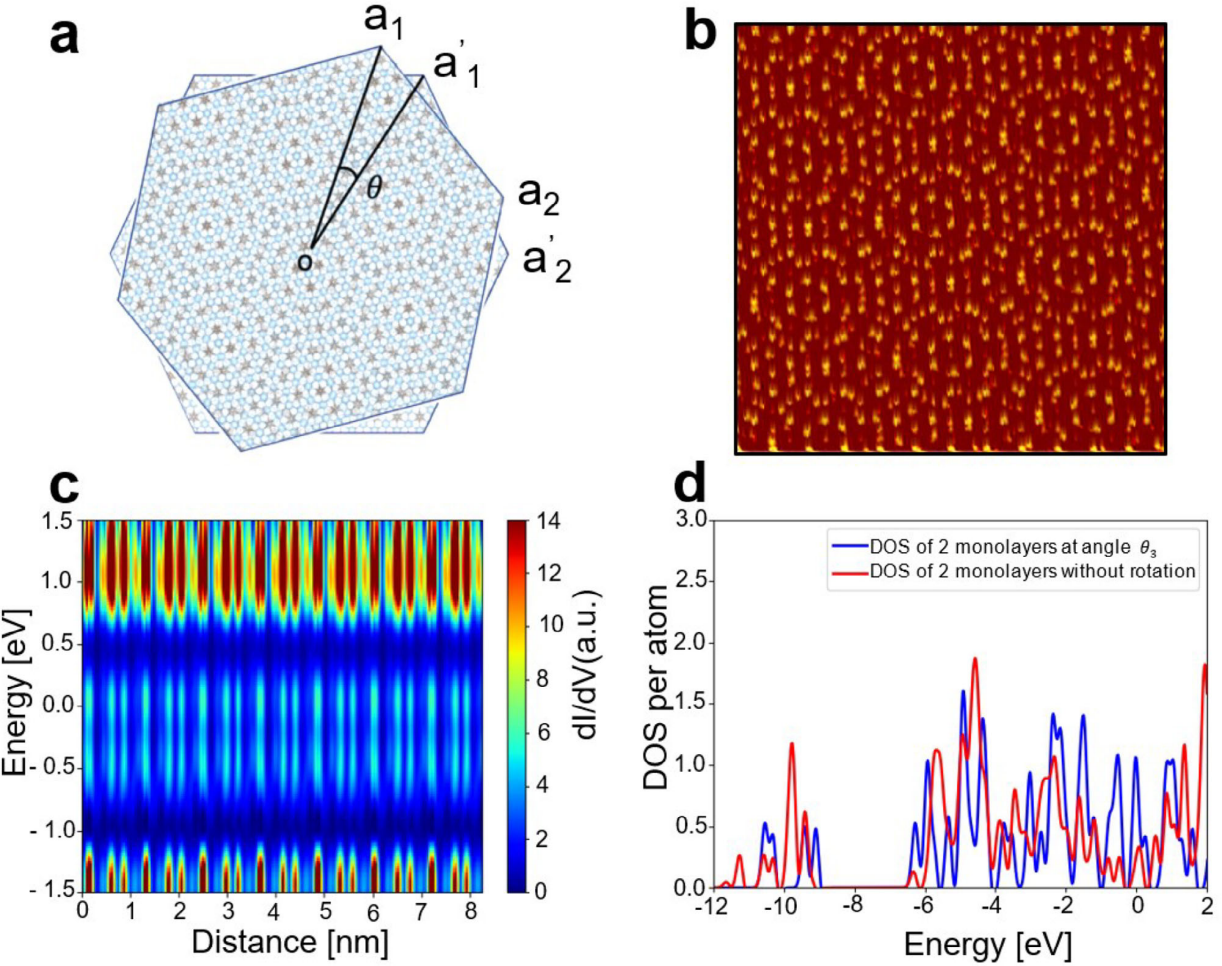
DFT simulation of STM, 2D STS, and DOS. a) Layer rotation of MXene with the model of the Moiré pattern at the *θ*_3_ angle. b) Simulated STM image for the MXene/MXene interface at the *θ*_3_ angle. c) Simulated 2D STS plot across the white line in Figure 3b. d) Comparison of the simulated total DOS at *θ*_3_ with the total DOS of two monolayers without any rotation.

Although Ti_3_C_2_T*_x_* MXene produced by wet‐chemical etching mainly shows three possible surface terminations (oxygen, fluorine, and hydroxyl groups), we utilized O terminations for our calculations, being the most abundant one (i.e., Ti_3_C_2_O_2_),^[^ ^43^ ^]^ as DFT calculations with mixed surface terminations have a much higher computational cost. The DFT‐STM simulation, along the same crystallographic direction as in Figure 3b, is shown in **Figure** **4**b, highlighting a clear Moiré pattern in good agreement with the experimental results. The DFT‐simulated 2D STS was computed along the white line in Figure 3b and can be seen in Figure 4c. Both the experimental and simulated 2D STS were taken over the ≈7 × 7 supercell (as the white line in Figure 3b passes through 7 supercells, from the high symmetry *X* point to the next high symmetry *X* point of the next supercell. Figure SM10 (Supporting Information) shows the 2D STS‐DFT along with the 7 supercells, with the 3 maxima marked for each supercell. Furthermore, in the simulated 2D STS, the exact physical positions of these 3 maxima along with corresponding atomic stacking configurations can be found in Figure SM11 (Supporting Information), which corresponds to three oscillations of the DOS within one supercell. In addition, the DFT simulation image shows spatial modulation on the conduction bands between 0.5 to 1.5 eV, which has a reasonable alignment with the experimental *dI/dV* results. However, simulations show a faint spatial modulation in the valence band as well, which is not detected in the experimental results. The presence of modulations in the LDOS from the DFT simulation, in alignment with the experimental STS, indicates that variation in interlayer coupling across the Moiré pattern,^[^ ^44^, ^45^ ^]^ as opposed to mechanical effects such as strain or local lattice relaxation, could be the primary physical mechanism responsible for the energy‐dependent modulation. Furthermore, to study the influence of the twist angle, the total DOS of two monolayers with no rotation and with a *θ*_3_ angle was compared in Figure 4d. Notably, the angle mismatch affects the total DOS and electronic structure as observed at the Fermi level, where a higher DOS compared to the total DOS with no rotation is observed, increasing the metallicity of the material. The twisted angle is large compared to typical “magic angles” where flat bands appear (e.g., 1.1° in bilayer graphene). For this system, the increase in DOS at the Fermi level is attributed to an increase in the availability of electronic states in the conduction bands, making the material more metallic,^[^ ^8^, ^46^, ^47^ ^]^ rather than the electronic flat bands as with the magic angle. Furthermore, the STS in this study shows no evidence of flat bands near the Fermi level, as other studies reported a peak structure at the Fermi level if flat bands were present.^[^ ^48^ ^]^ Additionally, Figure SM5b (Supporting Information) shows a comparison of the simulated total DOS of two monolayers at *θ*_3_ angle with respect to the total DOS of a monolayer without rotation, while Figure SM5c (Supporting Information) shows the comparison of the simulated total DOS of the infinite crystal at *θ*_3_ angle, decomposed into the orbitals’ contributions (black line), with respect to the total DOS of a monolayer (blue line). We see a significant change in the simulated DOS near the Fermi level induced by the Moiré pattern, indicating that the electronic structure is indeed heavily impacted by the introduction of the Moiré potential.

## 3 Conclusion

This work reports on the analysis and examination of self‐assembled Moiré superlattices in MXene using experimental scanning tunneling microscopy and spectroscopy techniques, supported by DFT calculations. We explored three distinct self‐assembled Moiré patterns with a periodicity of 2.52, 2.39, and 1.25 nm. Our STS results suggest a spatial modulation of the LDOS on the empty‐states side triggered by Moiré superlattice formation. This LDOS modulation is driven by underlying physical mechanisms, most notably electronic interlayer coupling, which induces local variations in the electronic structure across the Moiré superlattice. The experimental results were corroborated using DFT simulations, confirming that the electronic properties of MXene depend on the Moiré superlattice and the twist angle. This opens the door to applications of MXene in the field of twistronics. Furthermore, our findings verify that STM‐based techniques can be applied for targeted exploration of the atomic arrangements and electronic properties of the MXenes Moiré superlattice produced by an ad hoc deposition method. Finally, our results pave the way for further exploration of quantum phenomena and superlattices available for MXenes, expanding their already wide‐ranging portfolio of unique physicochemical properties.

## 4 Experimental Section

Ti_3_C_2_T*_x_* was synthesized from its parent MAX phase using the minimally intensive layer delamination (MILD) method.^[^ ^49^ ^]^ In a vented 40 mL PTFE vessel, deionised water (15 mL) was added, followed by drop‐wise addition of concentrated hydrochloric acid (45 mL, Sigma). To this, LiF powder (4.8 g, Sigma) was added, and the vessel was placed in an oil bath with stirring at 400 rpm using a PTFE stirrer bar for 10 min, to fully dissolve the LiF and allow the temperature to stabilize. Ti_3_AlC_2_ MAX phase powder (3 g, Carbon‐Ukraine, Ltd.) was then added in small portions to the vessel over a period of 15 min to avoid overheating of the solution. The solution was left stirring at 400 rpm at 35 °C for 24 h to obtain the etched, multilayer Ti_3_C_2_T*_x_* MXene. The contents of the vessel were then transferred evenly into two 50 mL centrifuge tubes and diluted to a total of 40 mL each with deionised water. The dispersion was then washed via centrifugation at 3000 RCF until the pH of the supernatant had reached at least 6.5. To delaminate the washed multilayer MXene, the tube was sealed tightly and shaken vigorously by hand for 30 min. The dispersion was then centrifuged at 1500 RCF for 30 min to sediment multi‐layer MXene or unreacted MAX phase. The supernatant containing delaminated MXene flakes was then collected. This dispersion was then centrifuged at 3500 RCF for 1 h to sediment the delaminated MXene, which was then redispersed to a concentration of ≈40 mg mL^−1^.

HOPG was cleaved in UHV. Subsequently, a solution of MXene with a concentration of 0.01 mg mL^−1^ was drop‐cast onto the freshly cleaved HOPG surface in a nitrogen environment, where the levels of oxygen and moisture were minimised. Following this, the sample was dried under vacuum conditions to remove any remaining oxygen present and no exposure to air. This meticulously controlled process ensures the adhesion of the MXene onto the HOPG substrate with minimum contamination. Afterward, the MXene/HOPG sample was subjected to mild annealing up to 200 °C for 24 h within the UHV environment (2 × 10^−10^ mbar). This process further ensures the removal of any remaining oxygen present and removes any absorbed water or contaminants. It should be noted that the chosen concentration of the MXene for the STM/STS studies was calibrated via atomic force microscopy measurements (Figure SM6, Supporting Information).

Atomic force microscopy (AFM) images were taken with a Bruker Dimension Icon microscope under ambient conditions, operating in tapping mode and using TESPA‐V2 tips with a spring constant k = 42 N/m. Images were captured at a scan rate of 1 Hz with 1024 lines per image.

For all STM/STS investigations, a low‐temperature STM from CreaTec was employed. The experiments were carried out under UHV conditions with a base pressure of ≈3 × 10^−11^ mbar. The STM images and STS spectra were acquired at a temperature of 77K using the constant current mode (CCM). Single‐crystalline W tips with a (001)‐orientation, prepared through electrochemical etching in NaOH, were cleaned in situ by Ar‐ion etching. The sample bias was maintained relative to the tip. During STS measurements, each spectrum was an average of 10 *I(V)‐*data points. The corresponding *dI*/*dV* spectra and 2D maps were obtained through numerical differentiation of plots derived from the *I‐V* spectroscopy. The STM tip height was stabilized through the CCM scanning parameters during movements.^[^ ^38^ ^]^ The STM preparation chamber was equipped with LEED, which provides information about the atomic arrangement and periodicity of the surface.

XPS spectra of the Ti 2*p* and C 1*s* core levels were recorded using an Omicron MultiProbe XPS instrument with a monochromatic aluminium X‐ray source (energy: 1486.7 eV) and an instrumental resolution of 0.6 eV, at a pressure of 8 × 10^−11^ mbar. After subtracting the Shirley background, the core‐level spectra were fitted using a combination of Gaussian‐Lorentzian line shapes with the CasaXPS software.

DFT calculations were performed by employing the Quantum ESPRESSO (QE) package, specifically the plane‐wave self‐consistent field (PWscf) code.^[^ ^50^ ^]^ This method requires periodicity to be able to simulate the Moiré pattern, and this can only be achieved at certain angles, which were summarized by M. Feuerbaher.^[^ ^51^ ^]^ In this case, the closest true periodic angle predicted by the Moiré lattice parameters is 13.17° instead of the experimental 11.9°.^[^ ^51^ ^]^ Furthermore, the pseudopotentials used for the simulations were LDA potentials from the GBRV library.^[^ ^52^ ^]^ The cutoff plane wave energy used for geometry relaxation was 40 Ry, as suggested in the literature for these potentials. For the calculation of the simulated STS, a higher cutoff of 60 Ry was used. The *k*‐point sampling for the monolayer calculations was 6 × 6 × 1. However, the *k*‐points were specified as gamma only for all the Moiré calculations due to the large size of the supercell. The initial cell parameters and atomic positions were taken from the Materials Project^[^ ^53^ ^]^ and were visualized by VESTA.^[^ ^54^ ^]^ The Moiré supercells were built from the relaxed atomic positions of the monolayer, without further relaxation of the ionic geometry. The STM images and STS curves were obtained using the Tersoff‐Hamman approximation.^[^ ^55^ ^]^

## Conflict of Interest

The authors declare no conflict of interest.

## Author Contributions

K.Z. and A.C.D.H. contributed equally to this work. The manuscript was written through the contributions of all authors. K.Z., A.C.D.H., and S.B. conducted STM, STS, and AFM analysis; A.C.D.H., S.B., D.D.O'R., and K.Z. performed theoretical calculations; D.S. and V.N. synthesized MXene; A.Z. conducted and analysed XPS measurements; K.Z. and V.N. conceived and designed the experiments, wrote the manuscript with key contributions from A.C.d.H. and S.B.; S.I. and Y.G. performed AFM measurements and assisted with interpreting the results and correcting the manuscript; I.V.S., Y.G., and V.N. helped with the planning of the experiments and supervision of the project. All authors discussed these results and reviewed the manuscript.

## Supporting information



Supporting Information

Supporting Information

## Data Availability

The data that support the findings of this study are available from the corresponding author upon reasonable request.
