# Supplementary material for: Self‐Assembled Moiré Superlattices of Ti3C2T x MXene for Future Twistronic Applications
Source: Adv Sci (Weinh). 2025 Jul 30;12(45):e04394. doi: 10.1002/advs.202504394 (PMC12677666; doi:10.1002/advs.202504394)
Supplement: Supplementary file 1 — Supporting Information [file ADVS-12-e04394-s002.docx]

**Supplementary Materials for**

**Self-assembled Moiré Superlattices of Ti_3_C_2_T*_x_* MXene for Future Twistronic Applications**

Kuanysh Zhussupbekov^1,2,§,*^, Andrea Cabero del Hierro^1,§^, Samuel Berman^1,^ , Dahnan Spurling^2,4^, Ainur Zhussupbekova^1,2^, Stefano Ippolito^3^, David D. O'Regan^1,4^, Igor V. Shvets^1^, Yury Gogotsi^3, ‡^ and Valeria Nicolosi^2,4,†^

*^1^ School of Physics, Trinity College Dublin, The University of Dublin, D02 PN40, Ireland*

*^2^ School of Chemistry, Trinity College Dublin, The University of Dublin, D02 PN40, Ireland*

*^3^ A.J. Drexel Nanomaterials Institute and Department of Materials Science and Engineering, Drexel University, Philadelphia, PA 19104, USA*

*^4^ Centre for Research on Adaptive Nanostructures and Nanodevices (CRANN) and Advanced Materials and Bioengineering Research (AMBER), Trinity College Dublin, The University of Dublin,* *D02 PN40, Ireland*

**1^st^ Method: Simulation method of the moiré pattern (equations)**

The following equation was used to calculate the angle mismatch for the interface between HOPG and MXene, where there is also a difference in lattice constant ^1^,

$\Delta=\frac{\left( 1-\delta\right)a}{\sqrt{2\left( 1-\delta\right)\left( 1-cos\phi\right)+ \delta^{2}}}$ (1)

where $\Delta$ is the periodicity (which can also be found represented by $L$), $\delta$ is the lattice mismatch between the topmost layers, $a$ is the lattice constant of the top layer, and $\phi$is the difference in angle. For MXene/MXene interfaces, the lattice constant is the same ($\delta=0)$, and the equation can be simplified to:

$L=\frac{a}{\sqrt{2\left( 1-cos\phi\right)}}$ (2)

while $1-cos\phi$ can be replaced by an expression in terms of $sin$ by using the following trigonometric equation

${sin}^{2}A=\frac{1}{2}\left( 1-\cos2A \right)$. (3)

Combining equations 2 and 3, we obtain the formula describing MXene/MXene interfaces:

$L=\frac{a}{\sqrt{2\left( {2sin}^{2}\frac{\phi}{2} \right)}}=\frac{a}{2 sin\left( \frac{\phi}{2} \right)}$ (4)

**2^nd^ Method: Simulation method of the moiré pattern (FFT)**


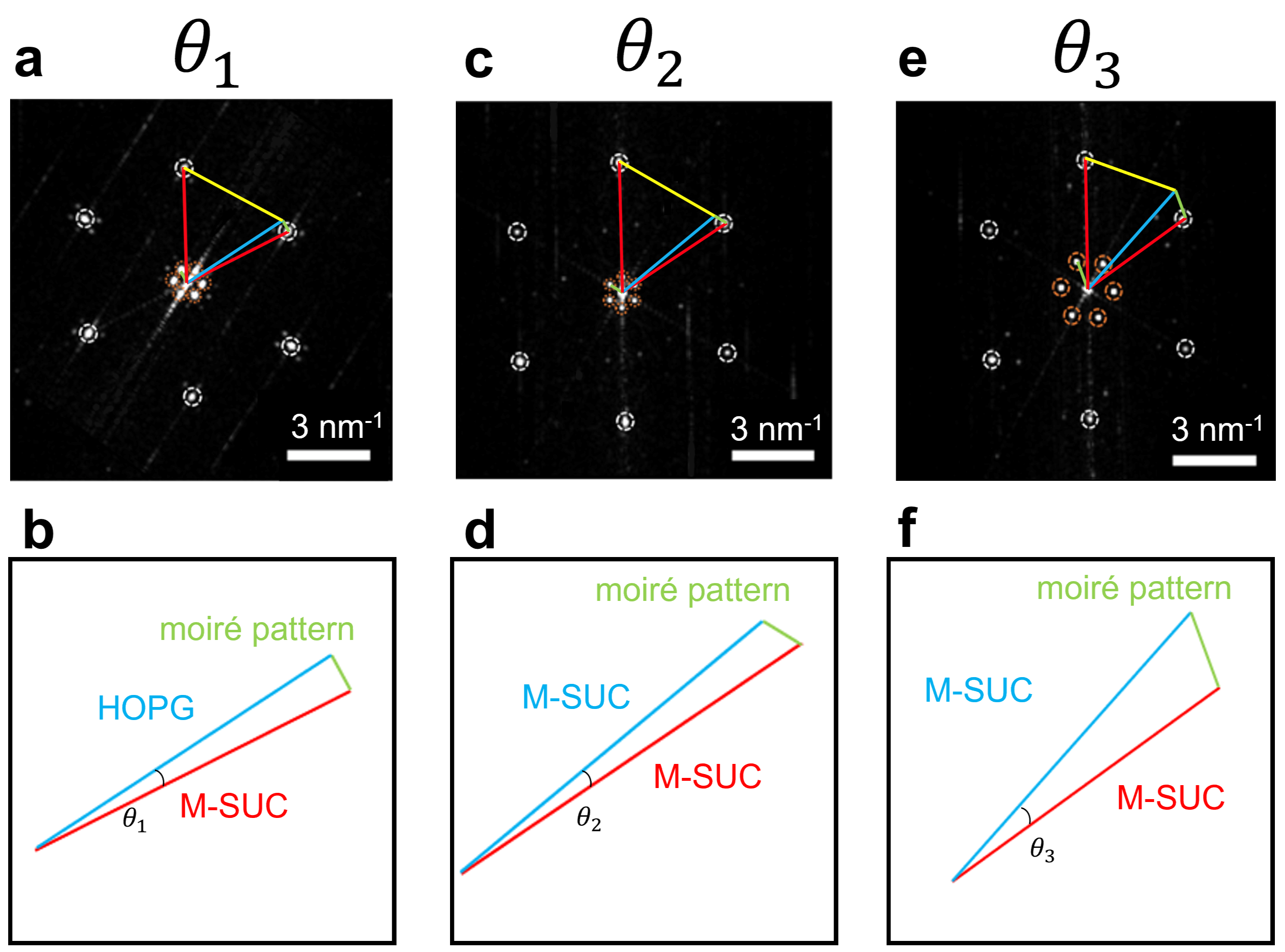


**Figure SM1.** **FFT method to find the twist angle of the moiré pattern. a, c** and **e** FFT of the STM topographic images in **Figure 2(c)**, **(d)** and **(e)**, where the blue line corresponds to HOPG in **a** and to M-SUC in **c** and **e**, the red line is M-SUC and the green line is the moiré pattern. **b**, **d,** and **f** Magnified schematic used to find the twist angle representing the wavevectors in reciprocal space.

In conclusion, using the schematics and the Law of Cosines, the angles were found to be *θ*_1_=6.0°, *θ*_2_=6.1°, and *θ*_3_=12.0°, in good agreement with the values of the angles found using the first method.


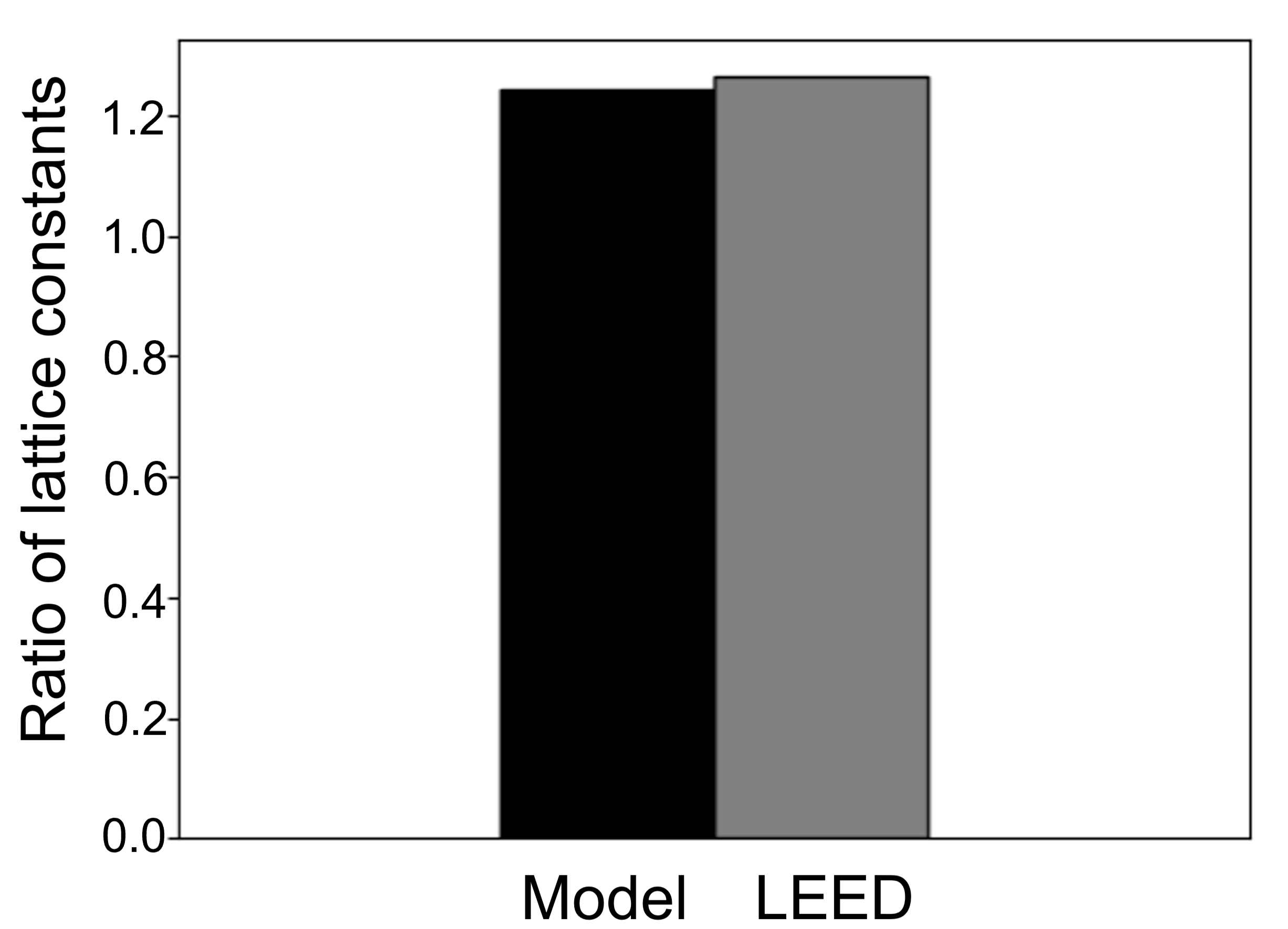


**Figure SM2. Comparison of the lattice constants.** The bar chart demonstrates the minimum difference between the ratio of lattice constants calculated from literature values and LEED patterns.


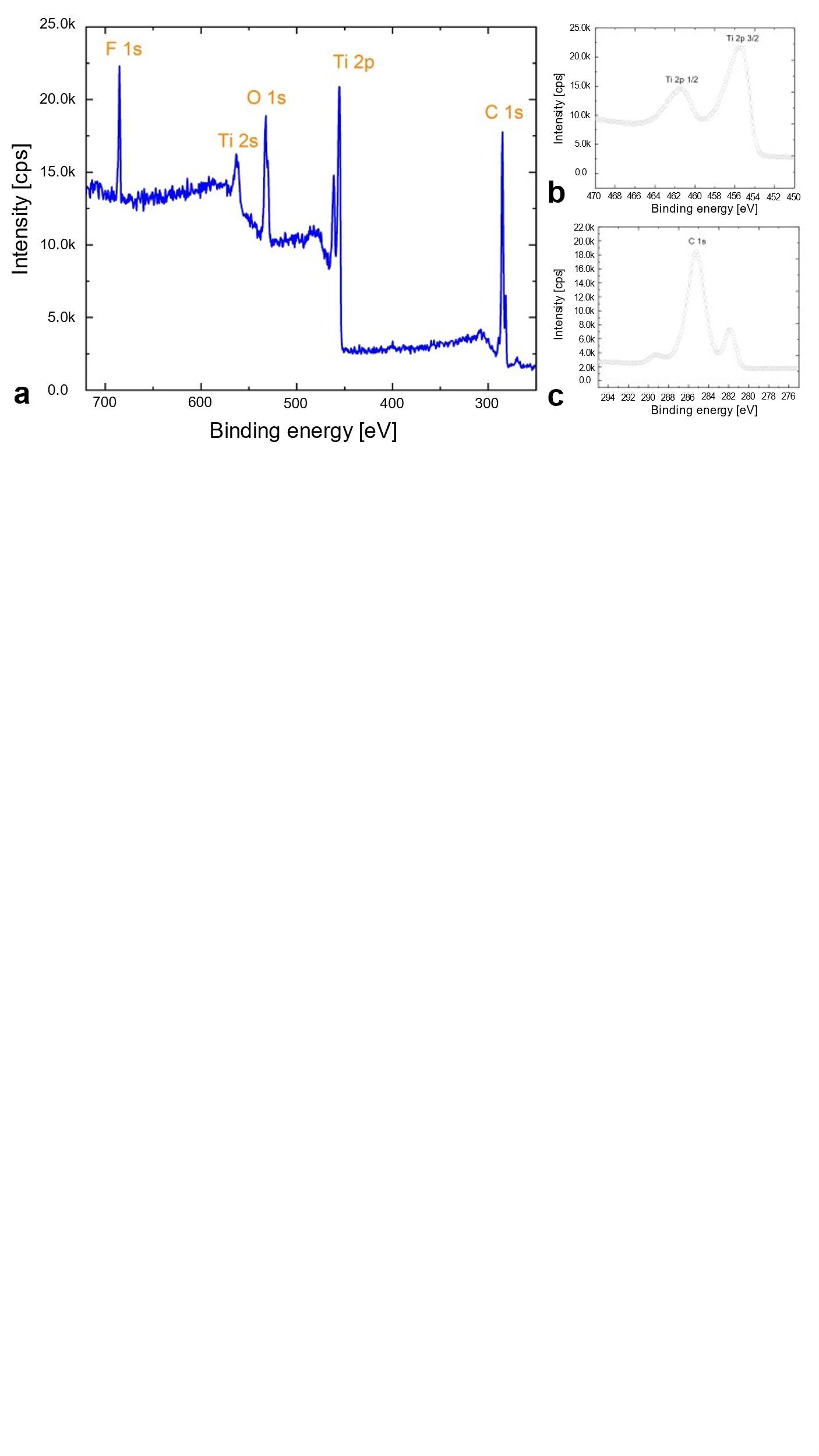


**Figure SM3. XPS of MXene from 1 mg/ml solution vacuum-dried on SiO_2_. a** Survey scan. **b** High-resolution Ti 2*p* region with fitted components. **c** High-resolution C 1*s* region with fitted components.


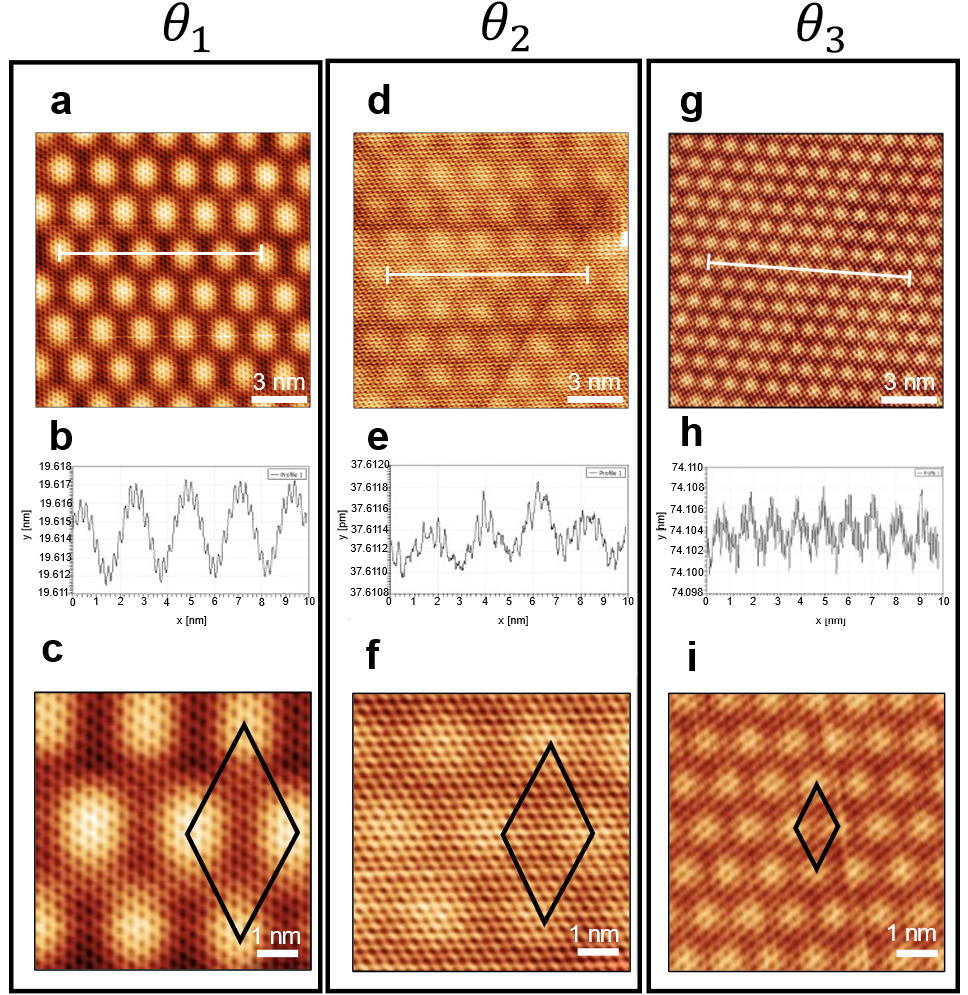


**Figure SM4.** **Moiré patterns**. **a** STM topographic image of the same area as in **Figure 2(c)** corresponding MXene/HOPG interface (15×15 nm, V = -1.2 V and I = 291 pA). **b** Corresponding line profile of **a**. **c** (7×7 nm, V = -1.2 V and I = 291 pA) where the black rhombuses indicates the superlattice of the pattern. **d** STM topographic image of the same area as in **Figure 2(d)** (15×15 nm, V = -1.2 V and I = 291 pA). **e** Corresponding line profile of **d**. **d** (7×7 nm, V = -1.2 V and I = 291 pA), where the black rhombuses indicates the superlattice of the pattern. **g** STM topographic image of the same area as in **Figure 2(e)**(15×15 nm, V = 1.5 V and I = 553 pA). **h** Corresponding line profile of **g.** **i** (7×7 nm, V = 1.5 V and I = 555 pA) where the black rhombuses indicate the superlattice of the pattern.


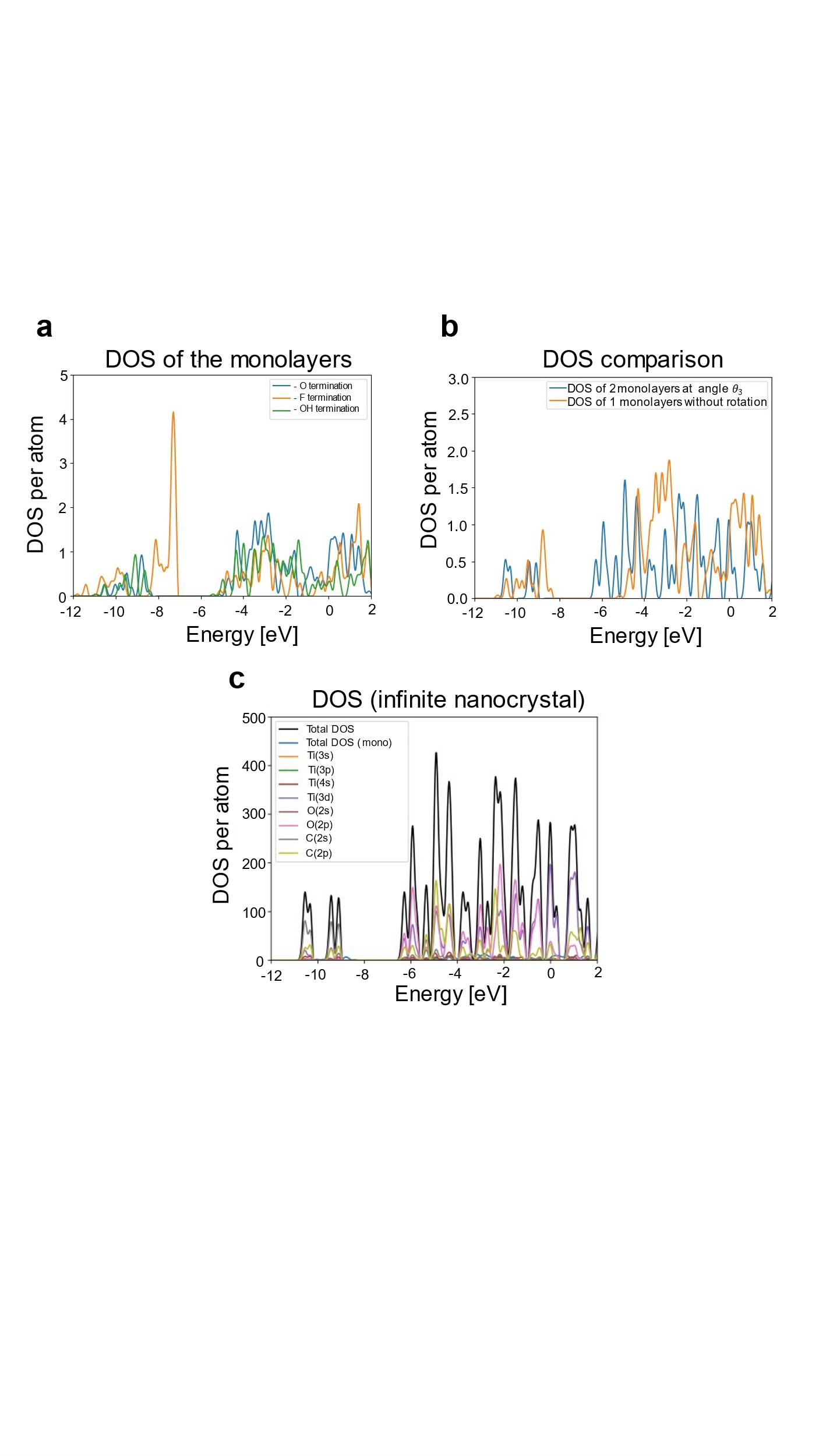


**Figure SM5.** **DFT-DOS calculations of monolayer and twisted MXene at *θ*_3_ degrees**. **a** Simulated DOS of the monolayers with the three possible terminations (-O, -OH and -F). **b** Comparison of the simulated total DOS of two monolayers at *θ*_3_ angle with respect to the total DOS of a monolayer (both cases with -O termination). **c** Comparison of simulated total DOS of the infinite crystal at *θ*_3_ angle, decompose into the orbitals’ contributions, with respect to the total DOS of a monolayer.


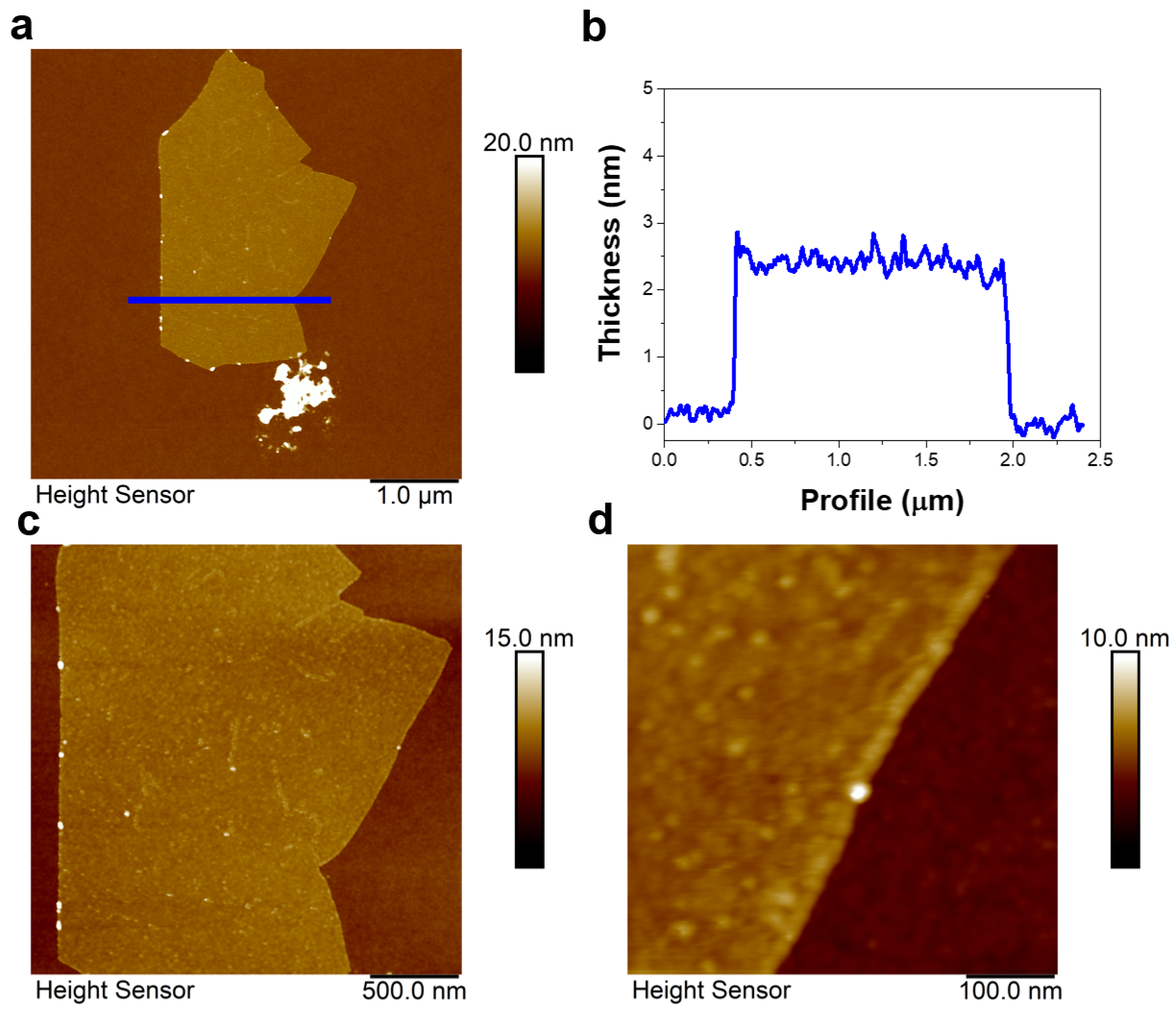


**Figure SM6.** **AFM characterization on ambient conditions**. **a** Large-scale AFM image of MXene on SiO_2_ substrate after sample preparation and being exposed to air for 3h. **b** Line profile of the corresponding blue line in **a**. **c** Zoomed AFM image in area **a**. **d** Zoomed AFM image of the area in **c**.


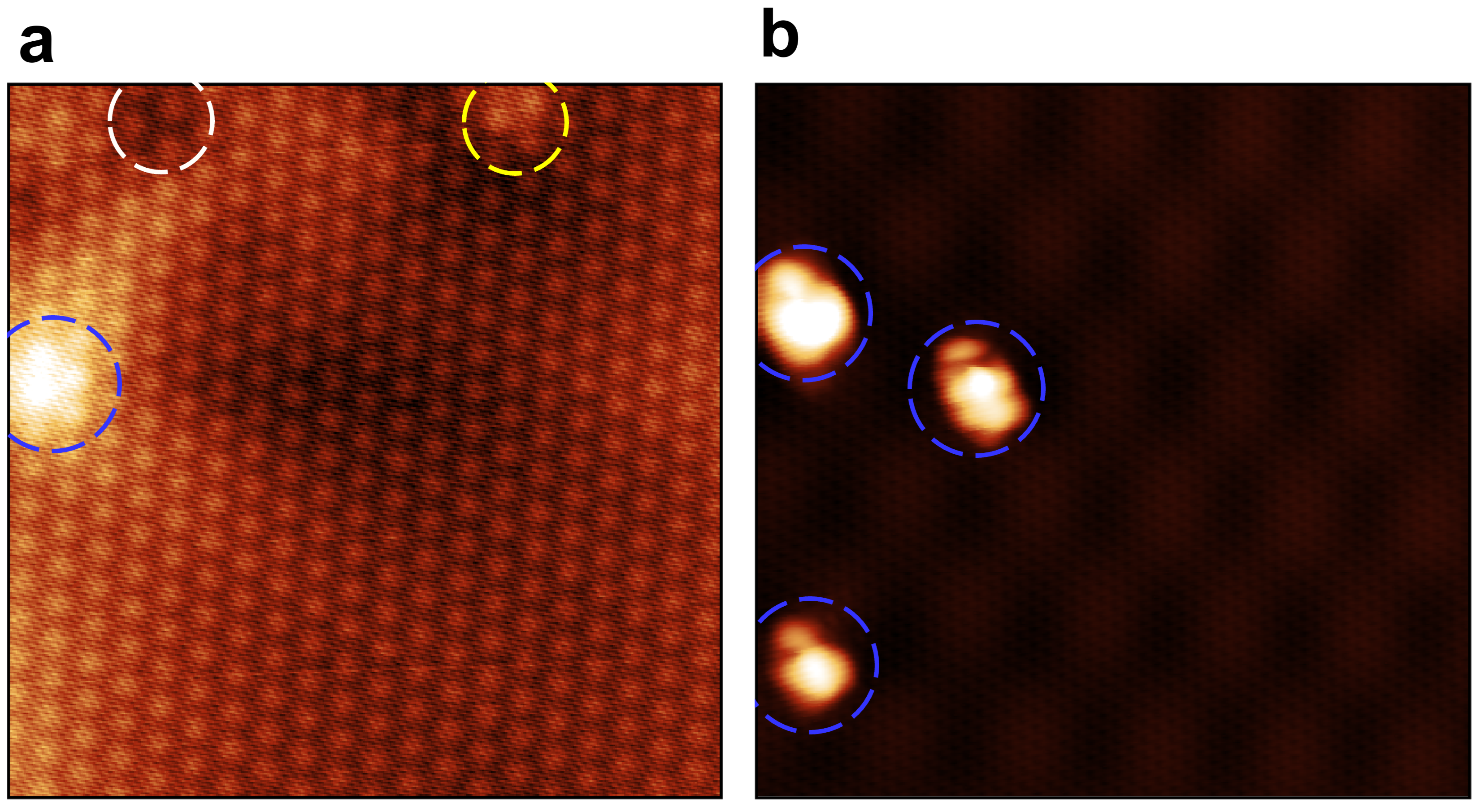


**Figure SM7.** **Defects and clusters on the surface of the MXenes**. **a** STM images of the point defects on the surface of the *θ*_3_ area. **b** Three clusters were observed on the surface of the *θ*_1_ area, which is consistent with earlier STM study.^2^


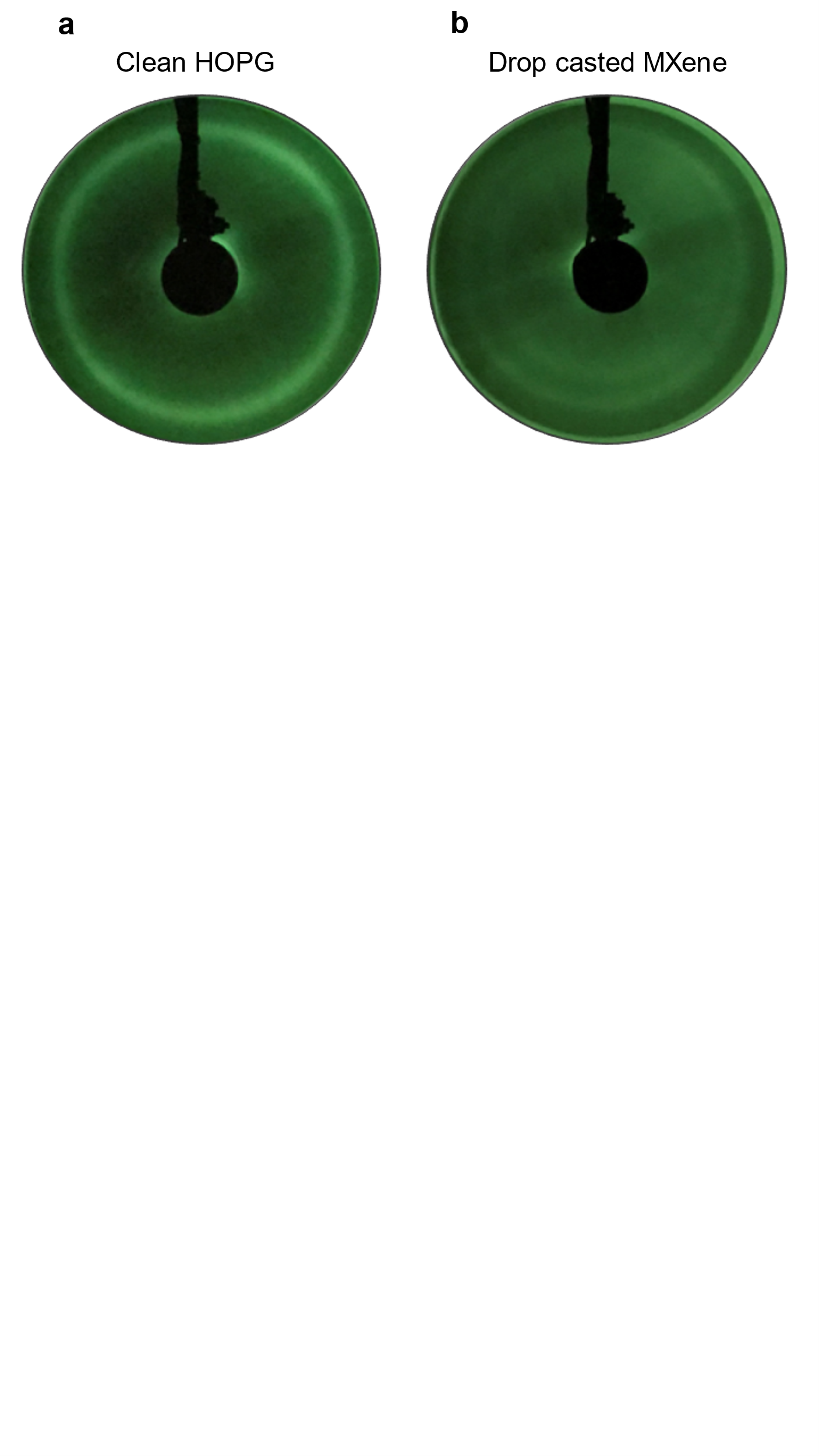


**Figure SM8.** **Raw LEED patterns of the HOPG substrate and drop casted and MXenes. a**, **b** Raw images of LEED pattern of HOPG surface cleaved in UHV and after drop-casting of MXene at a primary beam energy *E*_p_ = 118 eV.


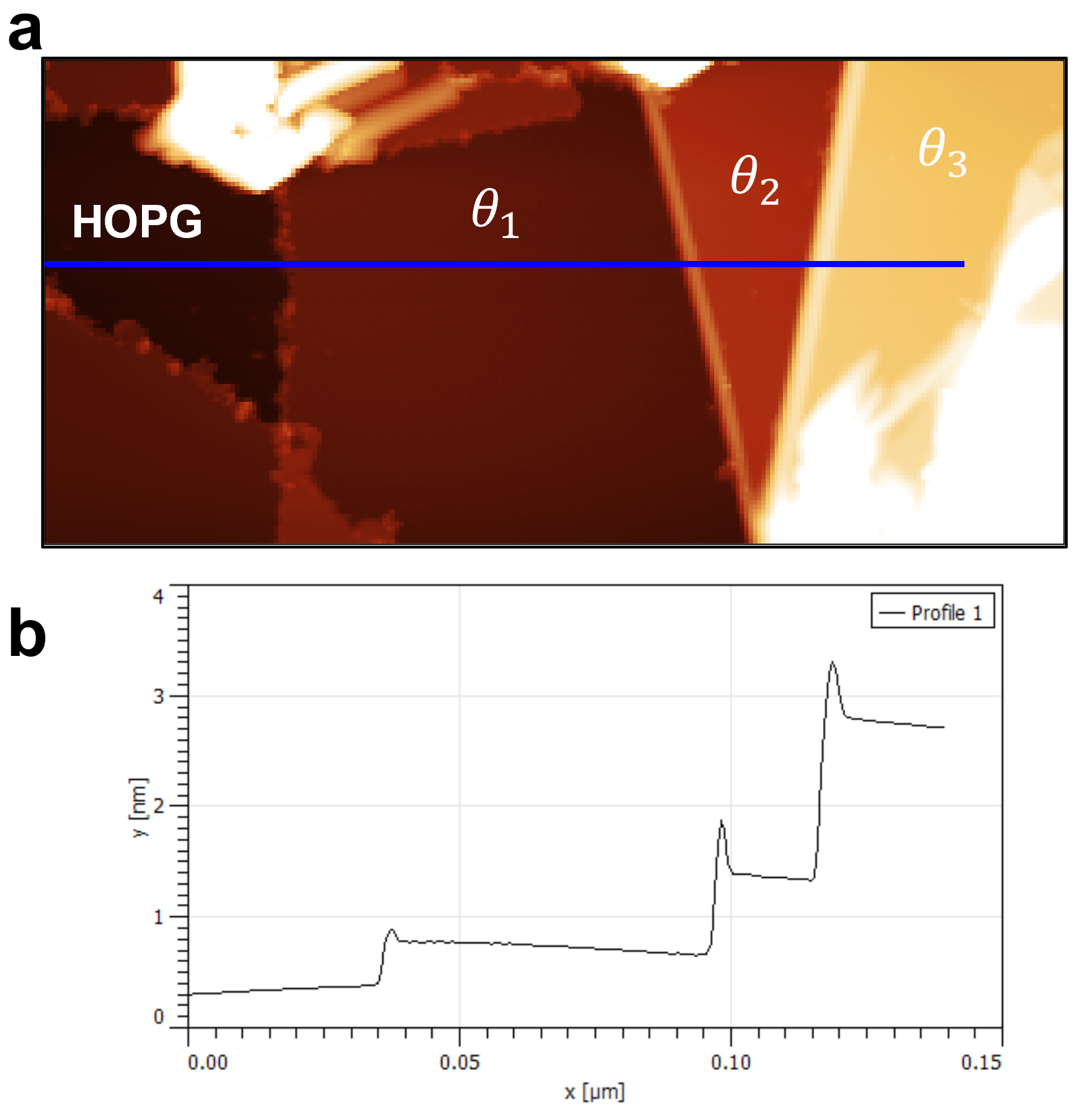


**Figure SM9.** **Large STM image where moiré areas were resolved with corresponding line .**

**a** Large-scale STM topographic image showing the MXenes area scanned (155 x 75 nm, V = - 1.2 V and I = -101 pA). **b** Line profile of the STM image in **a** indicated with blue line.


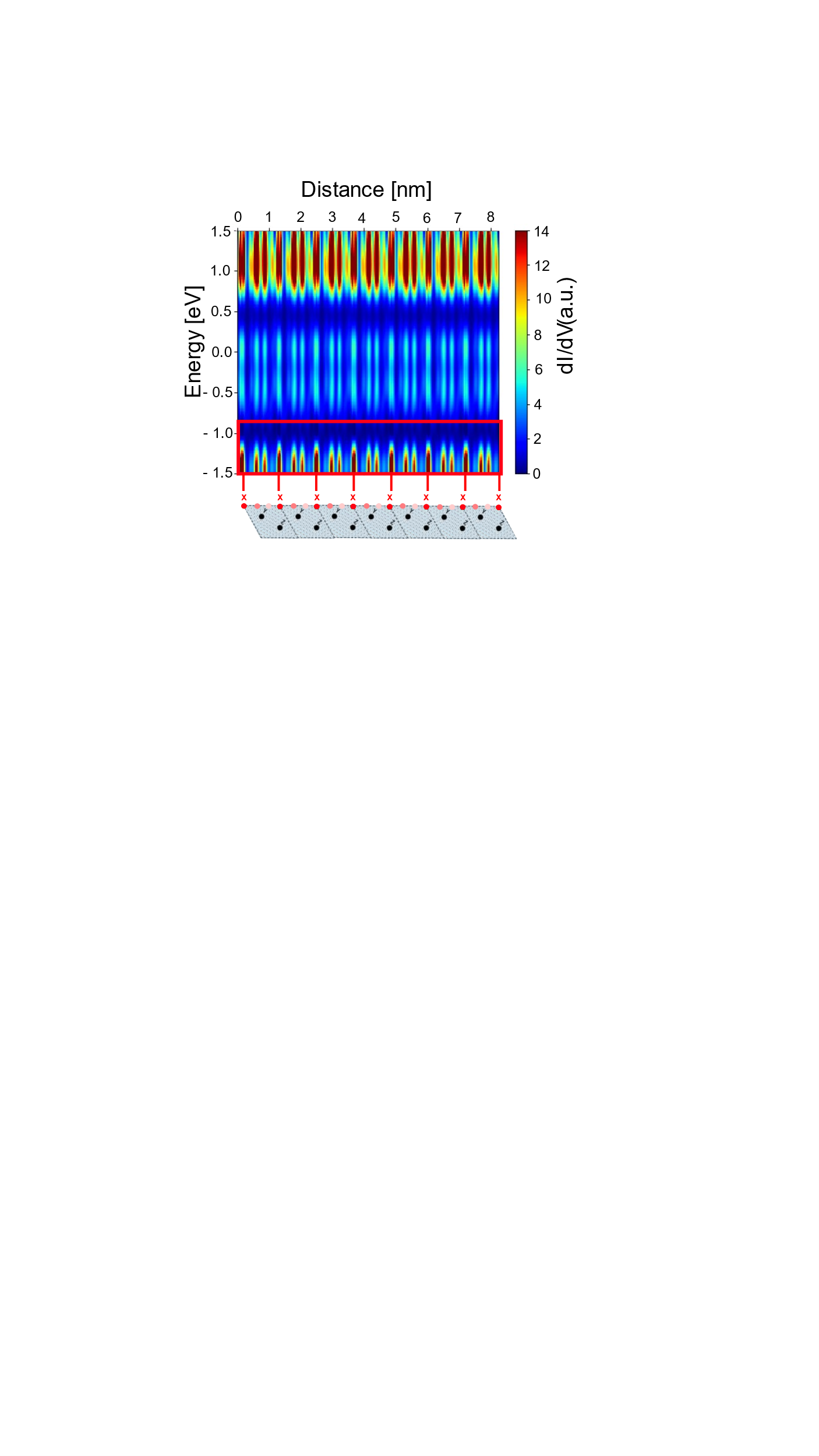


**Figure SM10.** **DFT simulation of STS along with the 7 supercells.** 2D STS-DFT simulation along the white line in Figure 3(b), which passes through 7 supercells from the high symmetry X point to the next high symmetry X point of the next supercell.


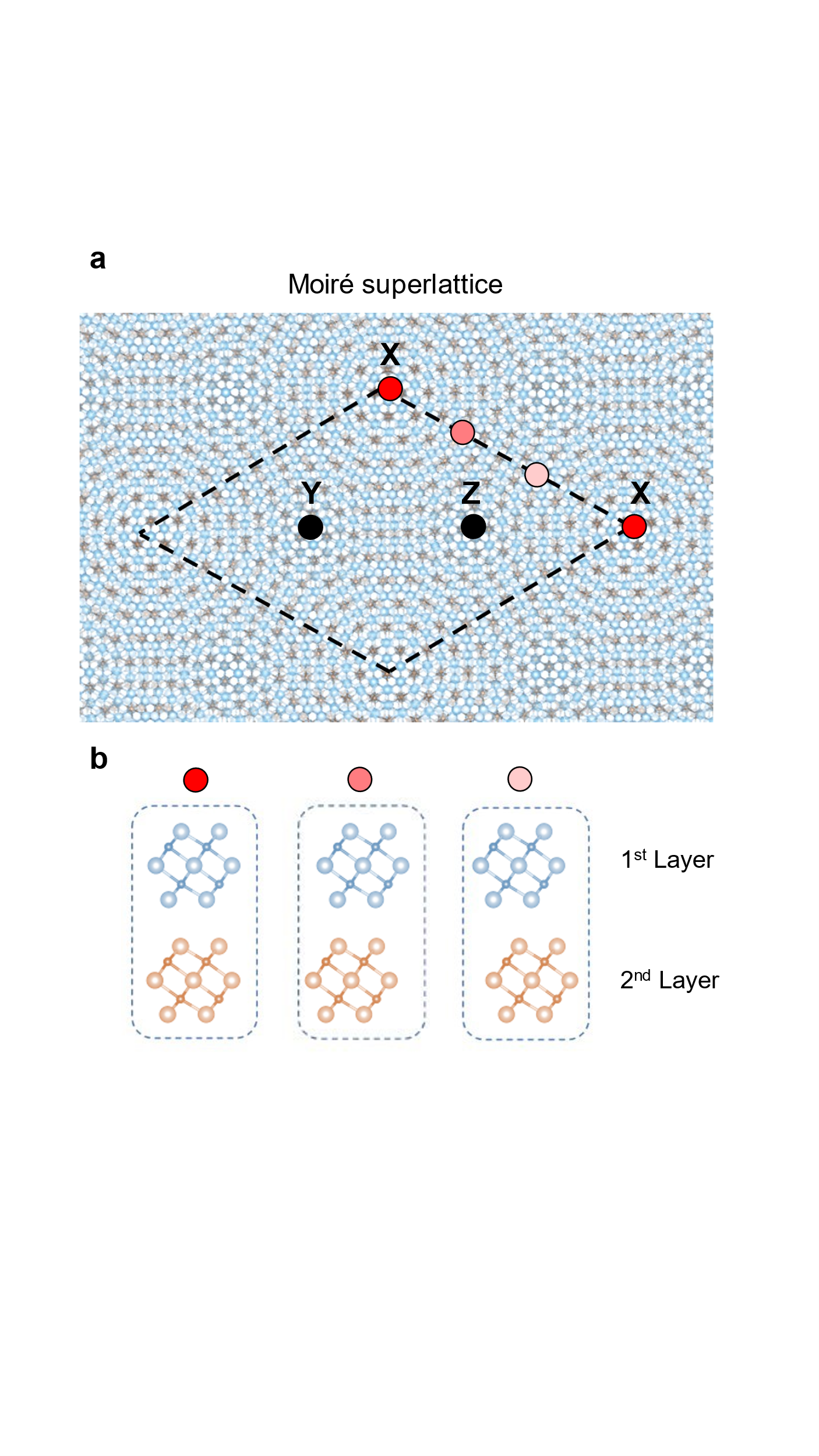


**Figure SM11.** **Schematic with physical positions of the maxima and its corresponding stacking configurations. a** Model of the simulated moiré pattern, showing the maxima stacking. **b** Alignment of MXene layers at the maxima, where large circles are titanium atoms and small circles are carbon atoms (shows the cross-section perpendicular to the surface of **a**.

**Bibliography**

1. Wang, J., Namburu, R., Dubey, M. & Dongare, A. M. Origins of Moiré Patterns in CVD-grown MoS_2_ Bilayer Structures at the Atomic Scales. *Sci. Rep.* **8**, 9439 (2018).
2. White, K. E. *et al.* Atomic-scale investigations of Ti_3_C_2_T*_x_* MXene surfaces. *Matter* **7**, 2609–2618 (2024).
